# The British Science Guild's Second Exhibition

**Published:** 1919-07-12

**Authors:** 


### SCIENCE IN INDUSTRY.

### The British Science Guild's Second Exhibition.

The exhibition of British scientific products held last year at King's College, under the auspices of the British Science Guild, proved so successful that it has been decided to make the exhibition an annual event. This year the Guild has secured more spacious and convenient premises for the display at the Central Hall, Westminster, where it was inaugurated on July 3, and is to remain open until August 5. In 1918 a "second edition" was held at Manchester, and it is to be presumed that the same course will be followed this year for the benefit of that or some other large provincial centre.

From last year's exhibition we learned of what had been done by science under the stress of war; this year, with the coming of peace, the lesson which the Exhibition should help to impress upon the nation is the invaluable service which science, properly utilised, can render in the building up anew of our national life and prosperity.

The exhibits this year are classified under eleven different headings, with a supplementary section for those received "too late for classification." There are two sections in which our readers will be specially interested—"Medicine and Surgery" and "Chemistry"—and both suggest that further real advance has been made by British firms in placing on the market, as the result of scientific effort, products and appliances which were formerly "made only in Germany."

### Medicine and Surgery.

The British Drug Houses, Ltd., show no fewer than 100 examples of drugs, etc., mostly manufactured in order to replace products of enemy origin. Many of the exhibits are identical chemically with well-known German specialities, such as Tannigen, Medinal, Migrainine, etc. The exhibit of Messrs. Burgoyne, Burbidges & Co., Ltd., consists entirely of medical and other chemical products of which the manufacture has been started since 1914 with the intention of replacing imports from Germany. In the exhibit of Messrs. Burroughes, Wellcome and Co., the principal place is given "Kharsivan" and "Neokharsivan"—two organic arsenical compounds to replace the German preparations so widely advertised as "salvarsan" and "neosalvarsan." As an annexe to this exhibit are illustrations of four of the more important investigations recently carried out at the Wellcome Chemical Research Laboratories. One of these is concerned with the "Resolution of Hyoscine and its hydrolytic products into their optically active components," a subject which has become important owing to the use of hyoscine in the treatment known as "twilight sleep." A number of surgical and diagnostic instruments recently introduced are shown by Down Bros., Ltd., including those for the direct transfusion of blood and electro-therapeutic treatment. An exceedingly interesting exhibit is that of the Telephone and Microphone Company, consisting of instruments made for use by the deaf from prescriptions prepared as the result of tests to ascertain the combinations of diaphragms, the volume of sound, and the form of sound-wave most suitable in each individual case. In the section devoted to electrical appliances, a demonstration is given of the optophone, a selenium instrument invented to enable the blind to read ordinary print by ear.

### Chemistry Section.

In most of the other sections something of special interest to our readers will be found. Thus in the Chemistry Section we are shown the filter papers produced since the war to supersede a German monopoly by the manufacturers of the famous "Whatman" drawing-papers. There are also numerous glass and other apparati for the laboratory made in England for the first time since the war. Until 1915 lanoline used in pharmacy as a basis for ointments in the cutaneous application of certain drugs was manufactured almost exclusively by Germany for the whole world; in that year the British Lanoline Company set to work to devise a British product of equal value, and how successfully will be seen in the samples it now exhibits. Similarly the Corticine Floor Covering Company proves that it has solved the problem presented by the pre-war German monopoly in the particular kind of compressed cork that was extensively used for various purposes under the name of "Suberite." Every one has heard of the "Ozonair" system of ventilation, but it is a revelation to find from the exhibit of Ozonair, Ltd., the many uses to which the principle of the system has been adapted, such as the sterilisation of water, the preservation of food, the treatment of suppurating wounds, and for inhalation as part of the medical treatment of various diseases.

A word or two should be said in acknowledging the excellence of the catalogue, which has been issued under the editorship of Sir Richard Gregory. It makes a volume of 331 pages, and, besides a description of the exhibits, contains a review of the scientific work carried out during the war of the Universities and other institutions, works research laboratories, and industrial research associations.

